# Computed tomographic lymphography for sentinel lymph node biopsy in male breast cancer: report of two cases

**DOI:** 10.1186/2193-1801-2-351

**Published:** 2013-07-29

**Authors:** Naoki Hashimoto, Yurie Kudo, Michihiro Kurushima, Yamato Suzuki, Takafumi Yachi, Tomohisa Tokura, Yutaka Umehara, Shinsuke Nishikawa, Kenichi Takahashi, Takayuki Morita, Fumiko Narita

**Affiliations:** Department of Surgery, Aomori Prefectural Central Hospital, Aomori, Japan; Nursing Department, Aomori Prefectural Central Hospital, Aomori, Japan

**Keywords:** Male breast cancer, Computed tomographic lymphography, Sentinel lymph node biopsy

## Abstract

Male breast cancer is rare, accounting for less than 1% of breast cancers. Because of its rarity evidence of the usefulness sentinel lymph node biopsy (SLNB) for male breast cancer has not been established. Moreover, a navigation system which can easily determine the incision site of SLNB is needed because a second incision for SLNB is necessary in most cases. We report successful computed tomographic lymphography (CTLG)-guided SLNB in two male breast cancer cases: the first patient was a 79-year-old man and the second was a 64-year-old man. Both had presented with a lump behind the nipple. Clinical diagnoses were early breast carcinoma in both cases. The second patient took tamoxifen 20 mg daily as neoadjuvant endocrine therapy. SLNs were clearly visualized by CTLG, allowing mastectomies with SLNB to be performed. Both SLNB were negative, such that axillary lymph node dissection was not needed. Preoperative CTLG is useful for visualizing lymph flow and detecting SLN in male breast cancer.

## Introduction

Sentinel lymph node biopsy (SLNB) has become a common procedure for breast cancer patients. The SLN concept was developed based on the finding that carcinoma cells draining into lymphatic vessels first reach specific (sentinel) lymph nodes. According to this theory, it is possible to avoid axillary lymph node dissection if SLNs are properly identified and thereby reduce the risk of postoperative complications, such as edema and numbness of the arm, is reduced (Kurosumi and Takei, 2007).

In SLNB, numerous methods for the injection of a radioisotope (RI) and/or blue dye have been reported, including peritumoral, intratumoral, subcutaneous, intradermal, and subareolar injections (Schwartz et al., 2002). However, these methods present certain disadvantages and potential pitfalls regarding SLN mapping. On the other hand, several studies on the application of indirect lymphography to obtain images of the lymphatic vessels and nodes were reported in the 1980s. Among them, Suga *et al.* identified SLN by three-dimensional multidetector-row computed tomographic lymphography (3D MDCT-LG) using a nonionic contrast medium (Suga et al., 2003). This technique may compensate for the disadvantages of the scintigraphic and blue dye-staining methods because clear visualization of the direct connection between the SLN and its afferent lymphatic vessels can be obtained on detailed cross-sectional anatomical images. Despite several papers to date having been published on computed tomographic lymphography (CTLG) in women, there have been no reported studies on men. Male breast cancer is a rare disease, accounting for <1% of all breast cancers and <1% of all annual cancer deaths in men (Fentiman et al., 2006). Because of the low number of affected patients, treatment for breast cancer in men has been extrapolated from treatment protocols for breast cancer in women (Gentilini et al., 2007). Since the incision for SLNB is necessarily separate from the primary surgery in men, a navigation system which can easily detect the incision site in the axillary region is required.

We identified the SLN in two male breast cancer cases employing CTLG and a dye-guided method and evaluated the usefulness of CTLG for SLNB in male breast cancer patients.

## Case reports

### Case 1

**Figure 1:**
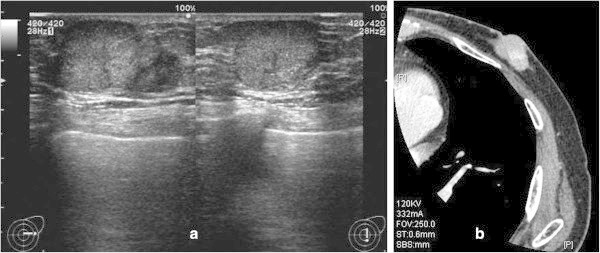
**Ultrasonography (a) and computed tomography (b) revealed a mass in the left breast with no enlargement of either axillary or cervical lymph nodes.**

A 79-year-old man was referred to our hospital with a 6-month history of a firm lump in his left breast. Ultrasonography (US) (Figure 1a) and contrast-enhanced computed tomography (CT) (Figure 1b) showed a mass in the left breast, but no enlargement of either axillary or cervical lymph nodes. Fine-needle aspiration cytology suggested malignancy. Tumor tissue obtained by core needle biopsy revealed invasive ductal carcinoma. There were no signs of distant metastases. Pre-operatively, the tumor was deemed T1N0M0 stage I, according to the TNM classification and staging criteria.

On the day before surgery, CTLG was performed to identify SLNs using a 64-detector row CT scanner. The patient was placed in the half side-lying position with the arms positioned in the cranial direction, essentially the same positioning as for surgery. To determine the vertical location of the SLN, a hand-made grid, consisting of 15 cm long angiographic catheters positioned at intervals of 1 cm and fixed together with adhesive tape, was placed on the lateral aspect of the anterior chest wall.

**Figure 2:**
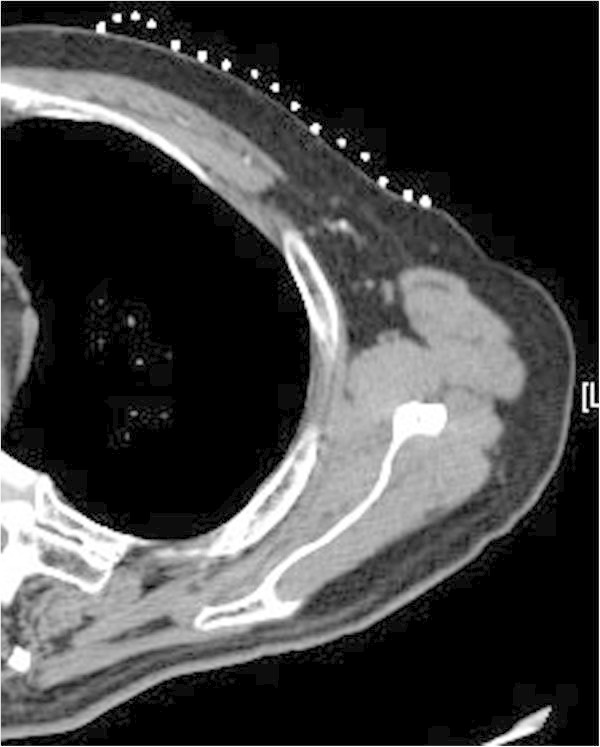
**Clear visualization of the direct connection between sentinel lymph nodes and its afferent lymphatic vessels can be obtained by computed tomographic lymphography.**

After induction of local anesthesia with a subcutaneous injection of 2 mL of 1% lidocaine hydrochloride, 4 mL of iopamidol were injected under the periareolar skin near the tumor using a 26-gauge needle. One minute later, CT images were obtained at a 2.5-mm slice thickness under operating condition of 120 kV, automatic current, a pitch of 0.969, and table speed of 19.37 mm/rod. SLNs were identified on axial images (Figure 2). The CT table was automatically moved to a suitable location, which was navigated according to the site of the SLN on the CT image. The SLN location was indicated precisely by the cross-point of the hand-made grid and horizontal lines of a thin beam of red laser light. Then, the location of the SLN was marked on the skin using an oil ink pen.

Under general anesthesia, 3 mL of indigocarmine were injected into the tumor side of the periareolar skin, followed by massage for 30 seconds. A 2 cm incision was made at the site marked with oil ink and the SLN was traced based on the lymphatic vessels dyed in blue. The removed sentinel nodes were immediately submitted to frozen section. After SLNB, total mastectomy was performed. Intraoperative pathological diagnosis showed no lymph node metastases, and axillary lymph node dissection was therefore deemed to be unnecessary. SLNs were fixed in formalin and pathologically examined. Thick tissue sections (2 mm in thickness) were cut from a paraffin block of SLNs. These sections were then stained with hematoxylin-eosin or immunostained with monoclonal antibody to cytokeratin.

Invasive ductal carcinoma was demonstrated pathologically. The tumor was histological grade 1, estrogen receptor (ER) positive, progesterone receptor (PR) positive, with a HER2 score of 0, and the Ki67 index was 15%. The SLNs showed no metastasis. Pathologically. The final diagnosis was pT1N0M0 Stage I. Hormone receptors were positive, and he received tamoxifen after surgery. The patient has experienced no recurrence, to date, for 3 years since surgery.

### Case 2

**Figure 3:**
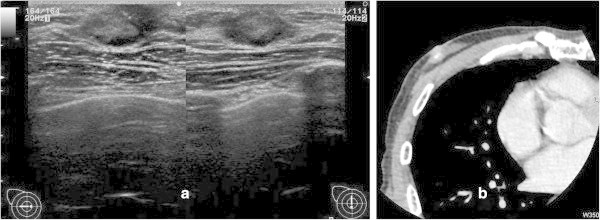
**Ultrasonography (a) and computed tomography (b) showed a tumor behind the nipple with neither node enlargement nor distant metastasis.**

**Figure 4:**
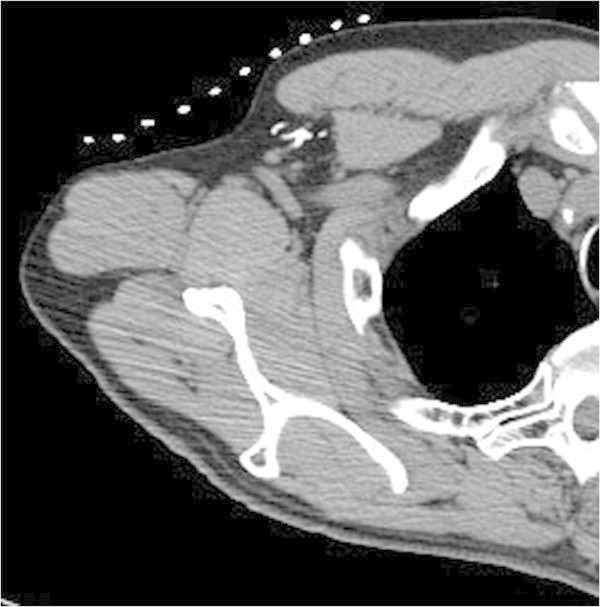
**Computed tomographic lymphography provided clear visualization of the lymphatic vessels and sentinel lymph nodes in the axillary region.**

A 64-year-old man visited a local clinic complaining of a 1.0 cm, well-defined, firm lump in his right breast that had been present for 7 months. He was referred to our hospital for further examination. US (Figure 3a) and CT (Figure 3b) revealed a tumor behind the nipple with neither node enlargement nor distant metastasis. Histopathological evaluation of the core needle biopsy material revealed invasive ductal carcinoma, ER(+), PR(+), HER2 score 0, and MIB-1 labeling index 20%. This patient was diagnosed as having T1cN0M0 stage I breast cancer. Because of his job, he could not undergo surgery immediately. He took tamoxifen 20 mg daily as a neoadjuvant endocrine therapy for half a year. Subsequently, a mastectomy with SLNB was performed. Before the surgery, CTLG provided clear visualization of the lymphatic vessels and SLNs in the axillary region (Figure 4). Histologically, the resected specimen showed invasive ductal carcinoma. The tumor was histological grade 2, ER(+), PR(+), with a HER2 score of 0 and Ki67 index of 10%. The histopathological response was moderate. The SLNs demonstrated no metastasis. Pathologically, the final diagnosis was pT1N0M0 Stage I. The patient has received adjuvant hormonal therapy (tamoxifen) with no signs of recurrence for 2 years.

## Discussion

Preoperative CTLG provides important information about the status of SLNs in patients with early-stage breast cancer. The relations among tumors, lymph-vessels, and SLNs can be assessed together with the surrounding anatomy. Guided by CTLG imaging findings, SLNB can easily be performed as a minimally invasive procedure. SLNB for breast carcinoma was introduced in the mid-1990s, and has already been performed on thousands of patients with breast carcinoma. Nevertheless, many unanswered questions remain (Schwartz et al., 2002). One of the problems to be solved is the lack of methods for identifying the full set of SLNs draining the primary tumor. As one possible solution to this problem, lymphangiography using an oil-in-water emulsion of contrast medium has been employed to assess the stages of malignancies. However, several complications have hampered widespread adoption of this method (Sato et al., 2007; Tangoku et al., 2004). Another possibility is CTLG, which allows visualization of the anatomy and provides functional data about lymphatic flow after interstitial injection of commercially available and commonly used intravenous contrast agents designed to enhance CT images. This method is simple, inexpensive and safe, such that CTLG has been performed in various fields, including video-assisted breast surgery (Yamashita and Shimizu, 2008), as well as in breast cancer patients requiring neoadjuvant chemotherapy (Ue et al., 2007) and patients with gastrointestinal malignancies (Tangoku et al., 2007). CTLG is technically easy to perform and the data acquisition time is short (Ue et al., 2007). In addition, CTLG can be performed at any institution equipped with a multi-detector row CT scanner.

In women, breast surgery is often performed using a lateral skin incision on the lateral mammary fold. This lateral skin incision saves the integrity of the skin blood supply, allows for a complete breast gland removal, and has benefits in terms of body image for women as no scars are apparent in the frontal view. Axillary clearance and/or SLNB can be easily performed via such a lateral skin incision (Regolo et al., 2008). On the other hand, the standard treatment for localized breast cancer in men is a modified radical or simple mastectomy including resection of the nipple since up to a third of men with breast cancer have stage III disease due to the small amount of breast tissue (Fentiman et al., 2006). For advanced male breast cancer, axillary dissection is usually performed via the same incision. However, in clinically node-negative cases, SLNB will probably become standard practice in the future. Another incision will thus be needed for SLNB in most cases. CTLG allows the detection of the sites of the SLNs preoperatively. Thus, the axillary region incision site can easily be determined, thereby shortening the operation.

CTLG will make SLNB in men with early-stage N0 breast cancer more practical, because CTLG has already become a standard method for women in many medical centers. Frequently, breast cancer is diagnosed in men at an advanced stage, making SLNB inappropriate, but a considerable proportion of patients still present with a clinically negative axilla, thereby making them candidates for a less invasive method of axillary staging (Gentilini et al., 2007). In addition, although the data were limited, a panel of the American Society of Clinical Oncology stated that SLNB would be unlikely to be any less accurate in men than in women, and that treatment of male breast cancer has paralleled that of female breast cancer (Lyman et al., 2005). The safety and usefulness of SLNB have also been reported for male patients. Thus far several papers have described SLNB in men, documenting detection rates of 100% and no false negatives in patients who received a back-up axillary clearance (Gentilini et al., 2007; Boughey et al., 2006; Cimmino et al., 2004; De Cicco et al., 2004; Flynn et al., 2008; Goyal et al., 2004; Kitada et al., 2011; Koukouras et al., 2012; Rusby et al., 2006). These findings support the reliability and reproducibility of SLNB as a technique applicable to male breast cancer patients.

In the American College of Surgeons Oncology Group Z0011 randomized trial, axillary lymph node dissection did not significantly affect overall or disease-free survival of patients with clinical T1-T2 breast cancer and a positive SLN who were treated with lumpectomy, adjuvant systemic therapy, and tangential-field whole-breast radiation therapy (Giuliano et al., 2011). More effective systemic therapy would presumably influence excellent local and distant outcomes, even if the regional nodes obtained by SLNB harbored metastases associated with a higher risk for systemic disease. A relatively large series of men with breast carcinoma suggested that men benefit from adjuvant systemic therapy for breast carcinoma, with the greatest benefit being that adjuvant hormonal therapy (Giordano et al., 2005; Ribeiro and Swindell, 1992). In our two cases, adjuvant endocrine therapy using tamoxifen was performed, and the second patient also received neoadjuvant endocrine therapy due to work-related issues. In men, CTLG has the potential to enhance the usefulness SLNB by reducing the complications associated with axillary lymph node dissection and improving quality of life with no reduction in survival duration.

In conclusion, CTLG allowed accurate SLN localization by providing rapid and adequate visualization of the direct connection between the SLN and its afferent lymphatic vessels. Detailed cross-sectional images of lymphatic anatomy during CT resulted in successful SLNB based on clear detection of the incision site. CTLG is especially valuable for identifying lymphatic drainage and the locations of SLNs in male breast cancer.

## Consent

Written informed consent was obtained from the patients for the publication of this report and any accompanying images.
